# Faulty risk-of-bias assessment in a meta-analysis of hydroxyethyl starch for nonseptic ICU patients

**DOI:** 10.1186/s13054-015-1080-9

**Published:** 2015-10-08

**Authors:** Ole Bayer, Konrad Reinhart

**Affiliations:** Department of Anaesthesiology and Intensive Care, Jena University Hospital, Erlanger Allee 101, 07747 Jena, Germany; Department of Anaesthesiology and Intensive Care, Center for Sepsis Control and Care, Chairman Global Sepsis Alliance, Jena University Hospital, Erlanger Allee 101, 07747 Jena, Germany

Evaluating 6 % hydroxyethyl starch (HES) of different molecular weight and substitution versus other fluids for nonseptic patients, He et al. [1] did not confirm the increased mortality, renal replacement therapy (RRT), bleeding and red blood cell transfusions seen in sepsis. A favourable safety profile for 6 % HES solutions was suggested.

He et al. stated that Preferred Reporting Items for Systematic Reviews and Meta-Analyses (PRISMA) guidelines were followed, yet the meta-analysis lacked tests of interaction to compare different HES solutions and pre-specified subgroup analyses. The discussed limitations of the meta-analysis included study quality, heterogeneity, and problematic use [2] of gelatin as a control fluid. The Cochrane Collaboration Risk-of-Bias Tool was cited for guidance in the assessment of study quality, where any use of scales for risk of bias is explicitly discouraged because summary scores have been shown to be unreliable. He et al. calculated summary scores using a nonvalidated modified Jadad scale [3] where the highest possible score is 8, but the highest possible summary score for He et al. was only 7; reasons for this difference are obscure. Information on how assessment of risk of bias was actually performed is missing. For selective outcome reporting, the study by James et al. was wrongly judged as ‘low risk’ although ‘high risk’ had been identified previously [4, 5].

The key message from He et al. that 6 % HES does not seem to increase mortality or RRT incidence in nonseptic ICU patients is speculative because assessment of the study quality is insufficient. HES should be considered unsafe in all ICU patients until proven contrary in high-quality studies.

## Abbreviations

HES: Hydroxyethyl starch
PRISMA: Preferred Reporting Items for Systematic Reviews and Meta-Analyses
RCT: Randomised controlled trial
RRT: Renal replacement therapy

## References

[1] He B, Xu B, Xu X, Li L, Ren R, Chen Z, Xiao J, Wang Y, Xu B (2015). Hydroxyethyl starch versus other fluids for non-septic patients in the intensive care unit: a meta-analysis of randomized controlled trials. Crit Care.

[2] Thomas-Rueddel DO, Vlasakov V, Reinhart K, Jaeschke R, Rueddel H, Hutagalung R, Stacke A, Hartog CS (2012). Safety of gelatin for volume resuscitation—a systematic review and meta-analysis. Intensive Care Med.

[3] Bañares R, Albillos A, Rincón D, Alonso S, González M, Ruiz-del-Arbol L, Salcedo M, Molinero LM (2002). Endoscopic treatment versus endoscopic plus pharmacologic treatment for acute variceal bleeding: a meta-analysis. Hepatology.

[4] Finfer S (2012). Hydroxyethyl starch in patients with trauma. Br J Anaesth.

[5] Reinhart K, Hartog CS (2012). Hydroxyethyl starch in patients with trauma. Br J Anaesth.

[6] Myburgh JA, Finfer S, Bellomo R, Billot L, Cass A, Gattas D (2012). Hydroxyethyl starch or saline for fluid resuscitation in intensive care. N Engl J Med.

[7] Annane D, Siami S, Jaber S, Martin C, Elatrous S, Declère AD (2013). Effects of fluid resuscitation with colloids vs crystalloids on mortality in critically ill patients presenting with hypovolemic shock: the CRISTAL randomized trial. JAMA.

[8] Zarychanski R, Abou-Setta AM, Turgeon AF, Houston BL, McIntyre L, Marshall JC (2013). Association of hydroxyethyl starch administration with mortality and acute kidney injury in critically ill patients requiring volume resuscitation: a systematic review and meta-analysis. JAMA.

[9] Mutter TC, Ruth CA, Dart AB (2013). Hydroxyethyl starch (HES) versus other fluid therapies: effects on kidney function. Cochrane Database Syst Rev.

