# Supplementary material for: Faulty risk-of-bias assessment in a meta-analysis of hydroxyethyl starch for nonseptic ICU patients
Source: Crit Care. 2015 Oct 8;19:357. doi: 10.1186/s13054-015-1080-9 (PMC4597439; doi:10.1186/s13054-015-1080-9)
Supplement: Related texts

# Reply: Authors’ response

**Authors:** Bin He, Bo Xu, Xiaoxing Xu, Lixia Li, Rongrong Ren, Zhiyu Chen, Jian Xiao, Yingwei Wang, Bin Xu

We thank Dr Bayer and Dr Reinhart for their comments, which we would like to nuance.

Recently, Myburgh et al. [6] reported that there was no significant difference in 90-day mortality between patients resuscitated with 6 % HES or saline. Another international multicentre large-sample randomised controlled trial (RCT) also reported the use of colloids (including HES, gelatin, dextran and albumin) versus any crystalloids for volume expansion therapy to decrease 90-day mortality [7]. In addition, two meta-analyses also showed that HES did not increase mortality or renal injury in subgroup analyses of nonseptic patients [8, 9]. Our meta-analysis also obtained similar results.

We had pre-specified subgroup analyses by different molecular weight of 6 % HES, risk of bias assessment and different kinds of fluids, all of which were found with no significant difference in mortality. We also made a brief analysis about the limitations of the meta-analysis. The Cochrane Collaboration Risk-of-Bias Tool and the modified Jadad scale were both always used for assessing the risk of bias [3, 8]. We apologise for the clerical error regarding the highest possible score in the modified Jadad scale and the inappropriate judgement for selective outcome reporting of the study by James et al.

Owing to the limitation of the current RCTs, our conclusion did not suggest using HES for these patients, and advised further RCTs of high quality for further study.
